# VabHLH137 promotes proanthocyanidin and anthocyanin biosynthesis and enhances resistance to *Colletotrichum gloeosporioides* in grapevine

**DOI:** 10.1093/hr/uhac261

**Published:** 2022-12-02

**Authors:** Dan Yu, Wei Wei, Zhongqi Fan, Jianye Chen, Yilin You, Weidong Huang, Jicheng Zhan

**Affiliations:** Beijing Key Laboratory of Viticulture and Enology, College of Food Science and Nutritional Engineering, China Agricultural University, Beijing 100085, China; State Key Laboratory for Conservation and Utilization of Subtropical Agro-bioresources/Guangdong Provincial Key Laboratory of Postharvest Science of Fruits and Vegetables, College of Horticulture, South China Agricultural University, Guangzhou, 510642, China; State Key Laboratory for Conservation and Utilization of Subtropical Agro-bioresources/Guangdong Provincial Key Laboratory of Postharvest Science of Fruits and Vegetables, College of Horticulture, South China Agricultural University, Guangzhou, 510642, China; State Key Laboratory for Conservation and Utilization of Subtropical Agro-bioresources/Guangdong Provincial Key Laboratory of Postharvest Science of Fruits and Vegetables, College of Horticulture, South China Agricultural University, Guangzhou, 510642, China; Beijing Key Laboratory of Viticulture and Enology, College of Food Science and Nutritional Engineering, China Agricultural University, Beijing 100085, China; Beijing Key Laboratory of Viticulture and Enology, College of Food Science and Nutritional Engineering, China Agricultural University, Beijing 100085, China; Beijing Key Laboratory of Viticulture and Enology, College of Food Science and Nutritional Engineering, China Agricultural University, Beijing 100085, China

## Abstract

Proanthocyanidins (PAs) and anthocyanins are involved in the response of plants to various environmental stresses. However, the mechanism behind defense-induced PA biosynthetic regulation is still not completely elucidated, also in grapevine. This study performed a transcriptome sequencing analysis of grape berries infected with *Colletotrichum gloeosporioides* to highlight the induction of the VabHLH137 factor from the basic helix–loop–helix (bHLH) XII subfamily by the fungus, which appeared to be significantly co-expressed with PA-related genes. The functional analysis of VabHLH137 overexpression and knockdown in transgenic grape calli showed that it positively regulated PA and anthocyanin biosynthesis. Moreover, VabHLH137 overexpression in the grape calli significantly increased resistance to *C. gloeosporioides.* A yeast one-hybrid and electrophoretic mobility shift assay revealed that VabHLH137 directly bound to the *VaLAR2* promoter, enhancing its activity and interacting with VaMYBPAR, a transcriptional activator of PA biosynthesis. Furthermore, transient experiments showed that although the VabHLH137 + VaMYBPAR complex activated *VaLAR2* expression, it failed to further enhance *VaLAR2* expression compared to VaMYBPAR alone. The findings indicated that VabHLH137 enhanced PA biosynthesis by activating of *VaLAR2* expression, providing new insight into the transcriptional regulation of defense-induced PA biosynthesis in grapevine.

## Introduction

Flavonoids are essential secondary metabolites in plants and are divided into several metabolic classes like isoflavonoids, flavones, flavonols, anthocyanins, and proanthocyanidins (PAs, also known as condensed tannins) according to their structures [1, 2]. In particular, PAs accumulate in leaves, stems, fruits, seeds, roots, and other parts of plants, depending on the plant species and the environmental conditions [2]. PAs play an essential role in plant growth and development and participate substantially in plant resistance to environmental stresses, including significant light exposure [3], UV irradiation [4], oxidative stress [5, 6], excessive temperatures [7], and pathogenic invasion [8–11]. In addition, PAs also influence the mouthfeel of agricultural products, such as fruits, wine, and beverages [12, 13]. As potential dietary antioxidants with various functions, PAs are widely considered beneficial to human health [14].

The biosynthetic flavonoid pathway has been extensively studied to isolate the corresponding structural genes in many plant species [15–19]. Leucoanthocyanidin reductase (LAR) and anthocyanidin reductase (ANR) are enzymes responsible for catalyzing the last specific steps in the PA pathway. Besides *Arabidopsis thaliana,* which only possesses the *ANR* (*BANYULS*) gene, most other plants contain at least one *LAR* gene in addition to the *ANR* gene. Although studies have revealed that *Vitis vinifera* contains two LAR orthologues with similar roles in controlling PA polymer biosynthesis [20, 21], the two genes exhibit different expression patterns during grape berry skin and seed development [20] and respond differently to stresses [4, 22]. Moreover, the *PtLAR3* transcription level is higher than that of *PtLAR1* in poplars with wounds or fungal infections [8]. Therefore, these results demonstrate differences in *LAR* genes regulation.

The enzymes involved in the flavonoid pathway control its overall efficiency and specificity [23]. Their genes are modulated by specific regulatory proteins, such as transcription factors (TFs), including MYB TFs, basic helix–loop–helix (bHLH, also known as MYC) TFs, WD40-repeat proteins, and MYB-bHLH-WD40 (MBW) complexes. Recent studies have identified the involvement of an increasing number of bHLH TFs in regulating the flavonoid pathway [24–29]. The *A. thaliana* and grapevine bHLH families contain 162 and 115 members, respectively [29, 30], and are classified into 26 phylogenetic groups in *Arabidopsis* [31]. The IIIf members of *Arabidopsis* are considered to be involved in flavonoid biosynthesis [32]. Grapevine VvMYC1, VvMYCA1, and VdbHLH37 belonged to the IIIf subfamily and were identified as flavonoid-related bHLH TFs that control anthocyanin and/or PA biosynthesis [29, 33, 34]. Furthermore, bHLHs often interact with different MYBs to regulate the separate branches of the flavonoid pathway, leading to flavonol, anthocyanin, and PA production [35]. For example, grapevine VvMYC1 activates anthocyanin and PA structural gene promoters by interacting with VvMYB5a, VvMYB5b, VvMYBA, and VvMYBPA1 [33]. However, the regulatory mechanism behind the involvement of other members of the bHLH family in PA biosynthesis remains unclear. In addition, bHLH proteins have demonstrated diverse functionality in response to stresses. Introducing MdCIB1 to apple calli and *Arabidopsis* improves drought stress resistance [36]. Moreover, MdbHLH33, in conjuction with MdMYBPA1, promotes anthocyanin production in apple calli in response to low temperature [37]. bHLHs are also essential for salt and iron deficiency tolerance [38, 39]. Recent studies involving grapevines, rice, and tomatoes have shown that fungal infection induces several bHLH TFs [40–42], implying that the bHLHs may be involved in biotic stress. However, the regulatory mechanism remains unclear.

Anthracnose is a fungal plant disease caused by the *Colletotrichum* species and can infect more than 3200 plants [43]. *C. gloeosporioides* is found in most viticultural regions in China and causes enormous economic losses [44, 45]. *Vitis amurensis* is an excellent wine grape variety known for its low-temperature tolerance and strong resistance to anthracnose [46]. This study characterizes the grape berry transcriptome in response to *C. gloeosporioides* infection to better understand *V. amurensis* to *C. gloeosporioides* resistance. *VabHLH137* is identified as differentially expressed upon infection and therefore functionally characterized based on the RNA-seq results. It participates in PA biosynthesis by binding to the *VaLAR2* promoter to activate its expression, interacting with VaMYBPAR, a PA biosynthesis activator. These results suggest that VabHLH137 is a novel PA biosynthesis regulator in the response to *V. amurensis to C. gloeosporioides*.

## Results

### *C. gloeosporioides* infection of the grape berries

**Figure 1:**
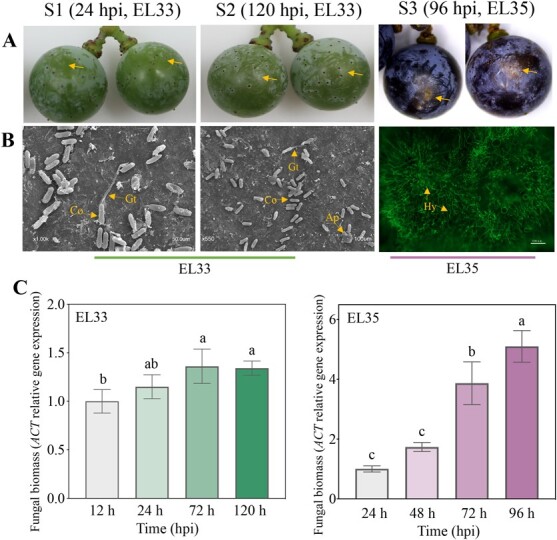
The *Vitis amurensis* cv. Zuoshan-1 berries infected with *Colletotrichum gloeosporioides*. The figure shows the grape berries at the EL33 and EL35 stages at the indicated time points after inoculation; in particular, 24 hpi and 120 hpi of berries at the EL33 stage (infection initiation and quiescent stage) and 96 hpi of berries at the EL35 stage (necrotrophic stage). **A** The asymptomatic inoculated berries at the EL33 stage (left and middle). The berries showing necrotic lesions at the EL35 stage 96 hpi (right). The arrows indicate the site of *C. gloeosporioides* inoculation. **B** The SEM of the conidia (co) germination and appressoria formation on the grape berries at the EL33 stage (left and middle). The fluorescence micrograph of the WGA-stained hyphae of the inoculated areas on the EL35 berries (right). Ap, appressoria; Co, conidia; Gt, germ tube; Hy, hypha. **C** The determination of the pathogen biomass via the relative gene expression of the *C. gloeosporioides* reference gene (*CgACTIN*) normalized against the *V. amurensis* reference gene (*VaActin*) expression during all inoculated berry stages. The means and standard errors were calculated using three biological replicates with 15 berries in each replicate. The different lowercase letters represent significant differences at *P* < 0.05 (Duncan’s test).

*V. amurensis* cv. Zuoshan-1 berries were inoculated with a conidial *C. gloeosporioides* suspension during the late green stage (EL33) and veraison stage (EL35). The disease progression was monitored for 120 hpi during the EL33 stage, presenting none of the expected infection symptom on grapes skin (Fig. 1A). Although microscopic observation showed a few conidia germinated germ tubes at 24 hpi and melanized appressoria formation within 120 hpi, but the colonization process on berry skins did not progress (Fig. 1B; Fig. S1, see online supplementary material). The appressoria and hyphae penetrated the cuticle and colonized the host, suggesting that *C. gloeosporioides* successfully proliferated on the grape berry surfaces. The fungal biomass further indicated the development of *C. gloeosporioides* on the berry skins, complementing the visual assessments (Fig. 1C). The *C. gloeosporioides* biomass slowly increased in a range from 12 hpi to 72 hpi at EL33 stage, indicating initial pathogen growth, followed by eventually cessation of the proliferation. Appressoria formation and the absence of an infection event suggested *C. gloeosporioides* quiescence on the berry skins. Fruit softening stimulates fungal transition from quiescent to necrotic growth. Diseased spots were observed on the surfaces of the berries at 96 hpi at EL35 stage, with long, thin hyphae marking the necrotrophic colonization (Fig. 1A and B). The fungal biomass increased quickly during EL35 stage (Fig. 1C), indicating rapid *C. gloeosporioides* proliferation in the grape tissues. Finally, the necrotrophic fungal colonization caused the berry skins to appear macerated.

### Transcriptome analysis and identification of VabHLH137 as a putative regulator of PA biosynthesis

To investigate the regulatory mechanism underlying the *V. amurensis* berry response to *C. gloeosporioides*, RNA-seq analysis was performed to compare the transcriptional changes in the infected and control berries at three selected time points, namely S1 (infection initiation, 24 hpi of EL33), S2 (quiescent stage, 120 hpi of EL33), and S3 (necrotrophic stage, 96 hpi of EL35).

**Figure 2:**
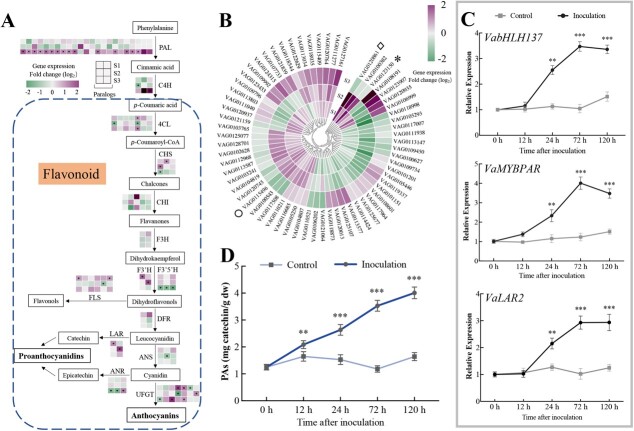
Different analyses for the identification of VabHLH137 as putatively involved in PA biosynthesis in *Vitis amurensis* cv. Zuoshan-1. **A** The flavonoid pathway modulation in the grapevine berries at three different stages upon *Colletotrichum gloeosporioides* infection. The fold change (log_2_) levels of the DEGs at the S1, S2, and S3 stages. **B** A heatmap showing the expression profiles of the TFs in response to *C. gloeosporioides*. The fold change (log_2_) levels of the DEGs at the S1, S2, and S3 stages. The asterisk represents *VabHLH137*, the circle represents *VaMYBPAR,* and the diamond represents *VaMYBPA1*. **C** The qRT-PCR analysis of the *VabHLH137* and PA biosynthesis-related genes in *V. amurensis* in response to *C. gloeosporioides*. *VvACTIN* and *VvGAPDH* genes were used for data normalization. **D** The PA accumulation in *V. amurensis* after *C. gloeosporioides* inoculation. Error bars represent the mean ± SD of three independent replicates. The statistical significance was determined using Student’s *t*-test (^**^P < 0.01; ^***^P < 0.001).

A total of 1326 (920 upregulated, 406 downregulated), 1505 (886 upregulated, 619 downregulated), and 1165 (737 upregulated, 428 downregulated) differentially expressed genes (DEGs) were identified between the infected and control berries at each time point (Table S2, see online supplementary material). Kyoto Encyclopedia of Genes and Genomes (KEGG) analysis revealed that the genes involved in flavonoid biosynthesis were strongly induced by *C. gloeosporioides* infection (Table S3, see online supplementary material). These genes included *VaLAR*, *VaANR,* and *VaUFGT*, which were responsible for PA and anthocyanin biosynthesis (Fig. 2A).

In addition, 64 differentially expressed TFs from 23 families were identified, including seven members of the MYB family and five of the bHLH family (Fig. 2B; Table S5, see online supplementary material). Of these, two positive PA biosynthesis regulators, *VaMYBPA1* (VAG0120861) and *VaMYBPAR* (VAG0115496) were strongly induced at both the S1 and S2 stages, confirming considerable PA accumulation in response to early infection. Moreover, the *VabHLH137* (VAG0123150) gene was upregulated during stages S1 and S2 and downregulated during stage S3, similar to the *VaMYBPA1* and *VaMYBPAR* gene expression profiles. The *VabHLH137*, *VaLAR2*, *VaMYBPA1,* and *VaMYBPAR* expression patterns in the grape berries at the EL33 stage were examined further via qRT-PCR after *C. gloeosporioides* infection (Fig. 2C; Fig. S3, see online supplementary material). The *VabHLH137* expression profile exhibited a significantly positive correlation with those of *VaLAR2*, *VaMYBPA1,* and *VaMYBPAR*, with Pearson’s coefficients of 0.96, 0.79, and 0.92, respectively. In addition, the *VabHLH137* expression profile was also consistent with PA accumulation in the green berries upon infection, which increased significantly, reaching a maximum level at 120 h (Fig. 2D). Furthermore, the induction of these PA biosynthesis-related genes and *VabHLH137* was accompanied by PA accumulation in the infected berries at the green stage. These results suggested the potential involvement of VabHLH137 in PA biosynthesis.

### Isolation and sequence analyses of *VabHLH137*

A phylogenetic tree of VabHLH137 with the reported bHLHs in grapevines and *Arabidopsis* was constructed via the neighbor-joining method using full-length amino acid sequences. VabHLH137 is closest to AtbHLH137 (AtCKG, AT5G50915) and belongs to the XII subfamily displaying a close relationship with AtCIBs (CRY2-interacting bHLH proteins), which all contained the bHLH_AtBPE_like domain and are clustered to the BEE/CIB clade (Fig. S4A, see online supplementary material).

To confirm the involvement of VabHLH137 in PA biosynthesis regulation in the grape berries, the CDS of *VabHLH137* was cloned from the *V. amurensis* cDNA library using gene-specific primers (Table S1, see online supplementary material). The *VabHLH137* sequence contained a 1050-bp open reading frame (ORF) that encoded a protein containing 349 amino acids. Multiple sequence alignments revealed that the predicted VabHLH137 proteins contained a conserved bHLH domain (Fig. S4B, see online supplementary material).

### Subcellular localization and transcriptional activity of VabHLH137

**Figure 3:**
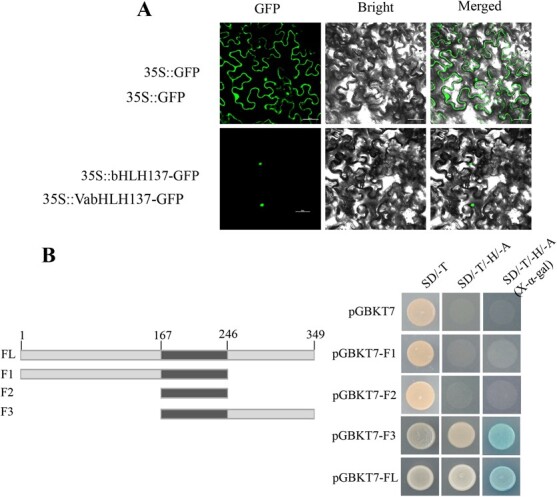
The subcellular localization and transcriptional activity of VabHLH137. **A** The localization of the VabHLH137-GFP protein in the tobacco (*Nicotiana benthamiana*) leaves. The GFP fluorescence signal of the VabHLH137-GFP fusion protein was observed in the nucleus. An empty 35S::GFP was used as a control. Bar = 50 μm. **B** Transactivation assay of VabHLH137 in the Y2H Gold yeast strain. Full-length (FL) VabHLH137 was divided into three fragments (F1–F3). The F3 fragment possesses transcription ability.

The subcellular localization of the VabHLH137 protein was investigated using a VabHLH137 recombinant plasmid with a green fluorescent protein (GFP) expressed in tobacco epidermal cells under the control of the CaMV 35S promoter. The VabHLH137-GFP fluorescent signal was exclusively localized in the nucleus, while the fluorescence of the control vector 35S::GFP protein exhibited a diffuse distribution and was present in both the cytoplasm and nucleus (Fig. 3A). The transcriptional activity of VabHLH137 was confirmed using a yeast-based transactivation assay (Fig. 3B). The yeast cells containing the full-length domain of VabHLH137 displayed excellent proliferation on synthetic dropout (SD) medium lacking tryptophan (Trp), histidine (His), and adenine (Ade) while showing α-galactosidase activity. These results indicated that VabHLH137 exhibited individual transcriptional activity. Furthermore, VabHLH137 was divided into three fragments according to its coding domain (F1-F3), showing that its C-terminal region of VabHLH137 possessed a transcriptional ability. Therefore, the data demonstrated that VabHLH137 acted as a TF in the nucleus.

### VabHLH137 binds to the *VaLAR2* promoter

**Figure 4:**
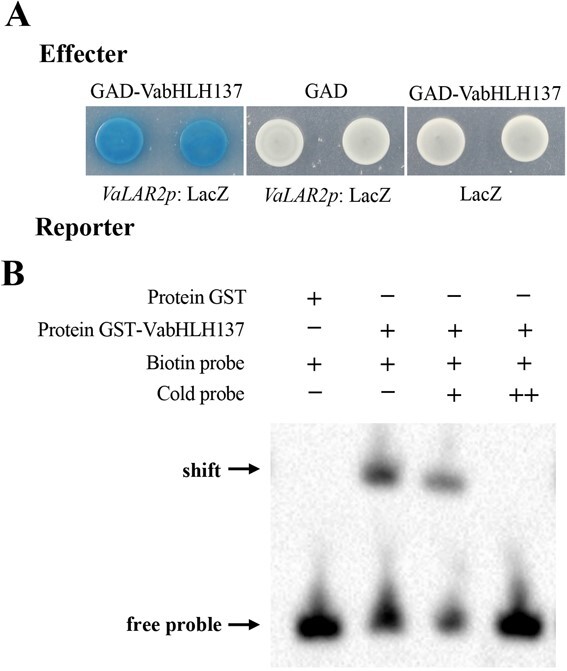
VabHLH137 binds to the *VaLAR2* promoter. **A** The interaction between the VabHLH137 protein and the *VaLAR2* promoter was determined via a yeast one-hybrid assay. The interaction between VabHLH137, fused to the GAL4 activation domain (GAD-VabHLH137), and LacZ driven by the *VaLAR2* promoter. The interactions were determined based on yeast cell growth and were confirmed by the color indication of X-α-Gal on an SD/−Trp/-Ura medium plate. **B** EMSA showing that the VabHLH137 could directly bind to the *VaLAR2* promoter. GST protein was used as a negative control. ‘−‘ and ‘+’ represent absence or presence, respectively. An unlabeled probe (cold probe) was used for competition. The arrows indicate the shifted band positions.

The *VabHLH137* expression profile correlated strongly with that of *VaLAR2* in the grape berries infected with *C. gloeosporioides* (Fig. 2C). A yeast one-hybrid (Y1H) assay was performed to determine whether the *VaLAR2* promoter was bound by VabHLH137. As shown in Fig. 4A, when AD-VabHLH137 was co-transformed with the *VaLAR2p: LacZ* reporter into yeast cells, VabHLH137 activated *LacZ* reporter gene expression, indicating that VabHLH137 directly bound to the *VaLAR2* promoter. The electrophoretic mobility shift assay (EMSA) showed that the GST-VabHLH137 fusion protein could bind to the E-box (CACATG) of the *VaLAR2* promoter (Fig. 4B).

### The overexpression of *VabHLH137* promotes PA and anthocyanin accumulation in the grape calli

**Figure 5:**
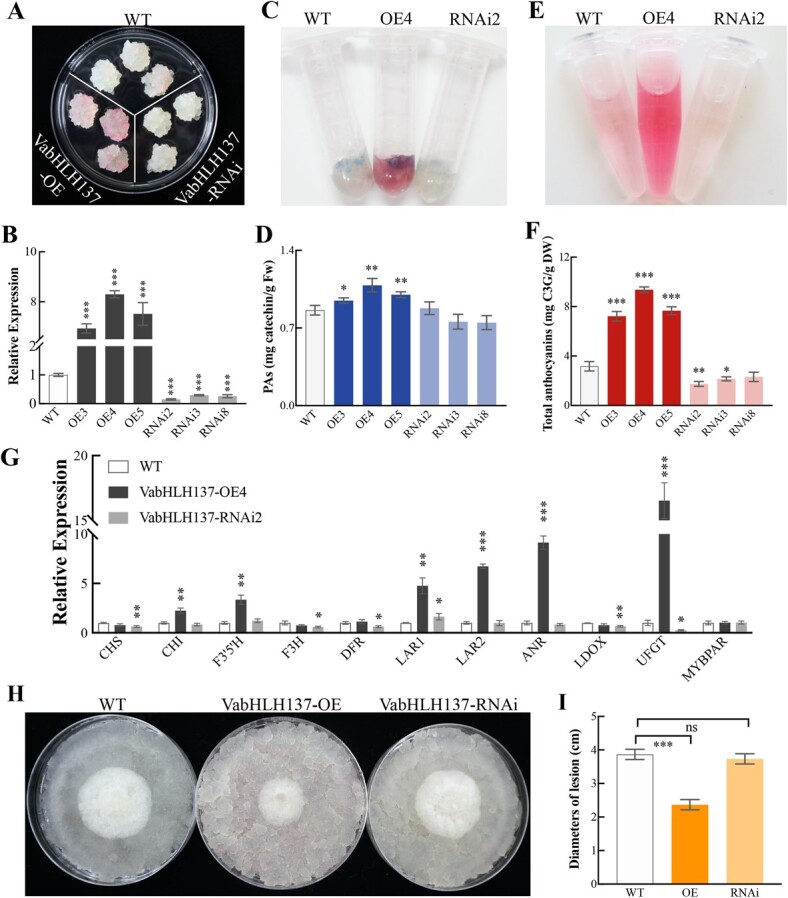
Functional characterization of the the *VabHLH137* overexpression in the grape calli. **A** The phenotypes of the transgenic grape calli. WT, wild-type grape callus; VabHLH137-OE, VabHLH137-overexpressing callus; VabHLH137-RNAi, VabHLH137-silencing callus. **B** The qRT-PCR analysis of the VabHLH137 expression level in the WT and transgenic grape calli. **C** DMACA staining of the WT and transgenic grape calli. **D** The PA content in the WT and transgenic grape calli. **E** The anthocyanins extracted in 1% (v/v) HCl-methanol. **F** The anthocyanin content in the WT and transgenic grape calli. **G** The transcript levels of the flavonoid-related genes in the WT and transgenic grape calli. *VvACTIN* and *VvGAPDH* genes were used for data normalization. **H** The representative phenotypes of the WT, VabHLH137-OE, and VabHLH137-RNAi transgenic calli infected with *Colletotrichum gloeosporioides* for 5 d. **I** The plaque areas in the WT, VabHLH137-OE, and VabHLH137-RNAi transgenic calli 5 d after inoculation with *C. gloeosporioides*. The statistical significance was determined using Student’s *t*-test (^*^P < 0.05; ^**^P < 0.01; ^***^P < 0.001).

To investigate the function of VabHLH137, constructs for *VabHLH137* overexpression and knockdown via RNA interference (RNAi) were introduced into *V. vinifera* Cabernet Sauvignon calli. After hygromycin screening for 3–5 generations, the stable overexpression and knockdown transgenic calli were obtained and identified via qRT-PCR. The transgenic callus (VabHLH137-OE) obtained via *VabHlH137* overexpression showed a visibly stronger red color than the wild-type (WT) control callus, while the RNAi (VabHLH137-RNAi) lines were whiter than the WT control (Fig. 5A). Moreover, because the reaction between dimethylaminocinnamaldehyde (DMACA) and the flavan-3-ol monomer and PA formed a blue chromophore [47], the callus was stained with DMACA reagent to visualize the PA level differences. Histochemical staining and spectrophotometric analysis revealed that the VabHLH137-OE lines produced substantially higher PA levels than the WT control, while the PA content of the RNAi lines did not change significantly (Fig. 5C and D). Quantitative measurements showed a substantial increase in the total anthocyanin content in the VabHLH137-OE callus, while a significant decrease was evident in the RNAi lines (Fig. 5E and F).

Furthermore, qRT-PCR analysis was performed to analyse the VabHLH137 transcriptional regulation of the flavonoid biosynthetic genes (Fig. 5G). The results showed that the genes involved in the flavonoid pathway, including *VvC4H*, *VvF3’5’H*, *VvLAR1*, *VvLAR2*, *VvANR*, and *VvUFGT,* were significantly upregulated during transgenic callus overexpression. Interference in the VabHLH137 expression in the callus markedly reduced the transcriptional *VvUFGT*, while PA structural gene expression (*VvLAR2* and *VvANR*) did not seem to be affected. These results demonstrated that VabHLH137 was involved in PA and anthocyanin biosynthesis.

### VabHLH137 improves the grape calli resistance to *C. gloeosporioides*

To confirm whether VabHLH137 could enhance plant resistance to anthracnose, the WT, VabHLH137-OE, and VabHLH137-RNAi calli were inoculated with agar plugs containing *C. gloeosporioides* hyphae. Furthermore, five days after inoculation, the plaque areas grown on the VabHLH137-OE transgenic calli were smaller than in the WT controls, suggesting that the *VabHLH137* overexpression in the grape calli increased fungal resistance (Fig. 5H and I). However, no significant differences were evident in the plaque areas between the VabHLH137-RNAi lines and WT control (Fig. 5H and I). This may be due to the lower anthocyanin level in the RNAi calli compared with the WT calli and minimal changes in the PA content, consequently exhibiting no differences in disease resistance.

### VabHLH137 interacts with VaMYBPAR

**Figure 6:**
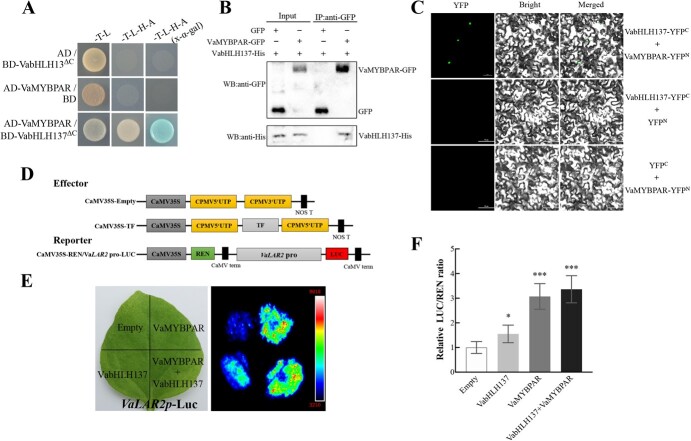
VabHLH137 interacts with VaMYBPAR. **A** The yeast two-hybrid assay revealing the interaction between VabHLH137^ΔC^ and VaMYBPAR. The yeast grown in SD/−Trp/−Leu medium and SD/−Trp/−Leu/−His/−Ade medium is indicated. X-α-Gal staining was used to further confirm the positive interactions. **B** CoIP assays showing the interaction between VabHLH137 and VaMYBPAR. The proteins were transiently expressed in the tobacco leaves. The VabHLH137-His proteins were immunoprecipitated with an anti-GFP antibody and immunoblotted with an anti-His antibody. **C** BiFC assay showing the interaction between VabHLH137 and VaMYBPAR *in vivo*. The epidermal cells of *Nicotiana benthamiana* were co-transfected with a mixture of *Agrobacterium* suspensions containing the VabHLH137–YFP^C^ with VaMYBPAR–YFP^N^ constructs. Green indicates a positive interaction signal. Bar = 50 μm. **D** The constructs used in the dual-luciferase reporter assay. **E** Detection of the LUC signal in tobacco leaves. **F** The effects of VabHLH137 and VaMYBPAR on the *VaLAR2* promoter activity, as demonstrated by the luciferase reporter assay. The values are means ± SD of six independent biological replicates. The statistical significance was determined using Student’s *t*-test (^*^P < 0.05; ^***^P < 0.001).

Based on the above evidence, it is possible to suggest that the VabHLH137 is involved in PA regulation. Then a yeast two-hybrid (Y2H) assay was performed to examine whether VabHLH137 formed a complex with VaMYBPA1 and VaMYBPAR to activate the *VaLAR2* promoter. Because the VabHLH137 full-length protein exhibited self-activation, a truncated VabHLH137^ΔC^ was inserted into pGBKT7 and co-transformed with the pre-harboring VaMYBPA1 or VaMYBPAR fusion protein. The results indicated that VabHLH137^ΔC^ interacted with VaMYBPAR (Fig. 6A) but not with VaMYBPA1 (data not shown).

A coimmunoprecipitation (CoIP) analysis was conducted to confirm the physical interaction. The two fusion proteins VabHLH137-His and VaMYBPAR-GFP were co-expressed in the tobacco leaves. The proteins were extracted and subjected to IP with an anti-GFP antibody. The IP fraction was analysed via a protein blot with an anti-His antibody. As expected, VabHLH137-His was detected in the bound protein via western blotting, confirming VabHLH137 and VaMYBPAR interaction *in vivo* (Fig. 6B).

Furthermore, a bimolecular fluorescence complementation (BiFC) assay was performed in tobacco leaves to test the VabHLH137 and VaMYBPAR interaction in planta. Fluorescent signals were observed in the nuclei of the tobacco cells co-transformed with VabHLH137-YFP^C^ and VaMYBPAR-YFP^N^ (Fig. 6C), while none were evident in the cells transformed with VabHLH137-YFP^C^ plus empty vector YFP^N^ or the empty vector YFP^C^ plus VaMYBPAR-YFP^N^. These results demonstrated a physical interaction between VabHLH137 and VaMYBPAR.

Finally, a transient expression assay was performed using the dual-luciferase system to further determine whether the VabHLH137 + VaMYBPAR complex activated *VaLAR2* transcription. The results showed that VabHLH137 and VaMYBPAR alone increased the *VaLAR2* promoter activity, with VaMYBPAR being more effective than VabHlH137. Moreover, the VabHlH137 + VaMYBPAR co-expressional activation of the *VaLAR2* promoter was almost similar to VaMYBPAR alone. The VabHLH137 + VaMYBPAR co-expression improved *VaLAR2* promoter activation (Fig. 6D–F).

## Discussion

As an effective chemical defense barrier, PAs accumulate during various biotic stresses. PA accumulated at the fungal infection site, reducing spore germination and mycelial growth [9, 11, 48]. In this study, the PA level in the grape berries increased significantly after *C. gloeosporioides* colonization (Fig. 2D). This defense-induced PA biosynthesis is controlled by the transcriptional abundance of structural genes and their TFs. bHLH TFs regulate plant responses to several environmental stresses and act as transcription activators or repressors to control PA biosynthesis [26, 27, 33]. To date, most of the reported PA-related bHLHs belong to the IIIf subfamily. However, minimal studies are available involving flavonoid pathway regulation by the other subfamilies. This work revealed a bHLH XII TF, *VabHLH137*, the expression of which in the immature infected berries was consistent with that of the PA-related genes (*VaLAR2*, *VaMYBPAR,* and *VaMYBPA1*) and PA accumulation. Although bHLH XII TFs reportedly participate in the development processes and response to abiotic and biotic stress in plants [36, 49–51], little is known about its involvement in stress-induced flavonoid biosynthesis. Several stress elements were identified in the *VabHLH137* promoter, including light-responsive motifs, wounding, and pathogen-responsive elements (Fig. S4, see online supplementary material), indicating that VabHLH137 could be widely involved in the response of *V. amurensis* to various stresses. The bHLH proteins regulated the transcription of stress-related genes by binding to the G-box (CACGTG) or E-box (CANNTG) elements in their promoter [52–54]. This study further demonstrated that VabHLH137 localized in the nucleus and bound the promoter region E-box of *VaLAR2* to induce its activity, suggesting that other bHLH subfamily members could regulate the flavan-3-ol pathway.

It has been shown that bHLHs, such as MtTT8 and CsNoemi, can slightly increase the PA level by activating PA-related gene expression [55]. Furthermore, light-induced *VvbHLH137* exhibits a similar expression pattern to the anthocyanin-related regulator *VvMYBA1* [56]. CabHLH137 binds to the *CaDFR* promoter in response to UV-B-induced anthocyanin biosynthesis in pepper [57]. In this research, *VabHLH137* overexpression in the transgenic grape calli promoted the gene expression involved in the PA and anthocyanin biosynthesis pathway, increasing the PA and total anthocyanin content and enhancing the resistance to *C. gloeosporioides* (Fig. 5H and I). However, *VabHLH137* overexpression produced higher anthocyanin levels in the transgenic calli than PAs. This may be because bHLHs are less specific than MYBs in regulating PA biosynthesis [58]. Additionally, PA and anthocyanin biosynthesis share the common upstream steps of the flavonoid pathway. *VabHLH137* significantly improved *VvUFGT* expression and competed for more substrates for anthocyanin synthesis (Fig. 5G). Moreover, the total anthocyanins decreased in the VabHLH137-RNAi lines, while no remarkable changes were evident between the PA content in the RNAi lines and WT calli, while the *VabHLH137* knockdown did not downregulate the transcriptional levels of the PA structural genes. Similarly, TT8 (AtbHLH42) positively regulated the *BAN* (*ANR*) in *Arabidopsis*. However, the *BAN* promoter activation was weak in the chalazal area of the developing *tt8* seeds [59], indicating that several bHLHs could regulate PA synthesis [60]. Although the result suggest that VabHLH137 is involved in defense-induced PA biosynthesis, it did not indicate whether VabHLH137 specifically regulates PA biosynthesis in response to *C. gloeosporioides* infection. Anthocyanin accumulation represents another common response of plants to multiple biotic and abiotic stresses [37, 61]. Here, *VabHLH137* overexpression boosted anthocyanin accumulation in the grape calli, while the anthocyanin content was too low to be measured in the infected grape berries at the EL33 stage. Therefore, whether VabHLH137-induced anthocyanin accumulation was involved in *V. amurensis* resistance to the early *C. gloeosporioides* infection could not be confirmed.

Previous studies involving various plant species have shown that MYB couples with bHLH partners to form regulatory complexes that synergistically activate PA biosynthesis gene expression [26, 33, 55]. Poplar PtMYB134, homologous to VvMYBPAR, was induced by fungal inoculation and interacted with PtbHLH131 to promote PA synthesis [11, 62]. In this study, VabHLH137 interacted physically with VaMYBPAR. However, the VaMYBPAR+VabHLH137 complex was not more effective than VaMYBPAR alone in activating the *VaLAR2* promoter. This could be attributed to the co-existence of the two transcriptional activators of PA biosynthesis. Their interaction may affect the formation of the MBW complex, influencing downstream target gene regulation [6]. Consequently, although interaction was evident between VabHLH137 and VaMYBPAR, they did not function as the reported complex.

In summary, the current work revealed that VabHLH137, a member of the bHLH XII subfamily, regulated fungal-induced PA biosynthesis in grapevines. The expression profile of VabHLH137 was similar to that of *VaLAR2* and *VaMYBPAR*, which were induced by *C. gloeosporioide*. Moreover, VabHLH137 interacted with VaMYBPAR and directly bound to the E-box in the *VaLAR2* promoter to activate its transcription, promoting PA accumulation and enhancing the resistance of *V. amurensis* to *C. gloeosporioide*. This study provided a new candidate gene to be tested in the complex PA regulatory network in further research. Future studies can investigate the modulating effect of VabHLH137 on the biosynthesis of other flavonoids and whether a bHLH TF from the IIIf subfamily forms an MBW complex with MYB TFs to regulate the PA pathway. Additional studies are needed to further elucidate the defense-induced flavonoid accumulation mechanism in grapevine.

## Materials and methods

### Plant material and growth conditions

The *V. amurensis* cv. Zuoshan-1 plants were grown in the grape germplasm resource repositories of the Institute of Botany, the Chinese Academy of Sciences (IBCAS) (39° 59′ N, 116° 12′ E). The grape skin-cultured calli of the *V. vinifera* cv. Cabernet Sauvignon, used for genetic transformation was cultivated on Gamborg’s B-5 Medium with 30 g/L sucrose, 2.5 g/L acid-hydrolyzed casein, 0.2 mg/L KT, 0.1 mg/L NAA, and 3 g/L phytagel (pH 5.9–6.0). The grape calli were incubated in a growth chamber at 25°C and a 16 h light/8 h dark cycle and sub-cultured at 15–20 d intervals. The *Nicotiana benthamiana* seedlings were grown in greenhouse at 25°C over a 16 h light/8 h dark cycle photoperiod.

### *C. gloeosporioides* inoculation

The modified Eichhorn-Lorenz system was used to collect the Zuoshan-1 berries at the EL33 and EL35 developmental stage for artificial inoculation with *C. gloeosporioides* [63]. About 20 berry clusters were randomly harvested during each developmental phase. Healthy berries were superficially disinfected with 70% alcohol (1 min), followed by 0.5% (v/v) NaClO (5 min), and rinsed four times with sterile water. The berries with pedicels were separated from the rachis and allowed to dry at room temperature for 1 h.

The *C. gloeosporioides* mycelia were cultured in potato dextrose agar (PDA, in Petri dishes) for 5 d at 28°C. The conidia were suspended in sterile distilled water and filtered through four layers of sterile gauze to remove the mycelia, after which the final concentration was adjusted to 1 × 10^6^ conidia/mL using a hemocytometer.

During EL33, 10 μl of the conidia suspension was dripped onto the grape berry surfaces using a pipette. To accelerate pathogen development from the quiescent stage to necrotrophic growth, and attain synchronous, uniform stage-specific colonization, the berries at the EL35 stage were punctured in three locations (each about 1 mm deep and 0.5 mm) with a sterilized needle and inoculated with 10 μl of the conidia suspension at each wound site. The control berries were similarly treated with sterile water. All treated berries were placed in plastic boxes at 95% relative humidity and stored at 28°C.

The infected and healthy (control) EL33 berries were collected at 24 h (S1), 72 h, and 120 h (S2) after treatment (hpi), while the EL35 berries were collected at 24 h, 48 h, 72 h, and 96 h (S3) hpi. Three biological replicates consisting of 30 berries were obtained at each sampling point. All berry samples were directly frozen in liquid nitrogen and stored at −80°C.

### Microscopic observation

Scanning electron microscopy (SEM) and wheat germ agglutinin (WGA) staining were used to observe the fungal development on the grape berries. For the SEM observation, exocarp segments (0.5 mm^2^) were excised from the inoculation points on the berries and fixed overnight at 4°C with 2.5% glutaraldehyde and 0.1 M phosphate buffer (pH 7.2). The samples were washed three times in 0.1 M phosphate buffer and dehydrated with a graded ethanol series (50%, 70%, 90%, and 100%). Subsequently, the samples were immersed overnight in isoamyl acetate at room temperature, subjected to critical point drying, and coated with gold for SEM (HITACHIS-3000 N, Hitachi, Japan) observation.

For the WGA staining, exocarp disks (1 mm^2^) were excised from the berries, fixed overnight with Carnoy’s solution at 4°C, and transferred to a 20% potassium hydroxide solution. The samples were washed three times with 0.1 M phosphate buffer (pH 7.5) and stained overnight with 20 μg/mL (g/vol) WGA buffer in the dark. Finally, the samples were washed three times in phosphate buffer and observed using a fluorescence microscope (Olympus BX-53, Tokyo, Japan).

### RNA extraction

The frozen samples were ground using a mortar and pestle, and the seeds were removed carefully by hand. Total RNA was isolated from the infected and control samples using the RNAprep Pure Plant Plus Kit (Polysaccharide&Polyphenolics-rich) (Tiangen, Beijing, China) according to the protocol of the manufacturer. The samples were then treated with the Recombinant DNase I. The concentration and quality of the RNA samples were determined using a Nanodrop 2000 spectrophotometer (Thermo Scientific, Waltham, MA, USA) and Agilent Bioanalyzer 2100 system (Agilent, Santa Clara, CA, USA), respectively. RNA integrity was also confirmed using 1% agarose gel. High-quality RNA was used for RNA-seq and quantitative PCR.

### RNA-seq analysis

A total of 18 samples were analysed via RNA sequencing by performing three replicates for each sampled time point (S1, S2, and S3). The paired-end reads of 150 bases were obtained using the Illumina HiSeq 4000 platform (Illumina, San Diego, CA, USA). The Illumina sequence reads were pre-processed by removing low-quality and adapter sequences, and clean reads were aligned independently with the V1 version of the *V. amurensis* reference genome [64] (http://www.grapeworld.cn/ggh/amu.html). Cufflinks v2.1.1 was used to assemble the transcripts and calculate the fragments per kilobase of transcript per million mapped reads (FPKM). Data normalization and the identification of the DEGs were performed using DESeq2. Genes with a log_2_ fold change ratio > 1 and *q*-value <0.05 were considered significantly differentially expressed between the treated and control samples. The functional annotation of DEGs was performed using Blast2GO. The Gene Ontology (GO) and KEGG enrichment analyses were performed using the TBtools software [65]. The RNA-seq data were deposited in the NCBI Sequence Read Archive (SRA) under the accession number PRJNA705401.

### Quantitative real-time PCR (qRT-PCR)

The qRT-PCR assays were performed using the LightCycler® 480 instrument II (Roche Life Science, Switzerland) with SYBR Green Master Mix (Transgen Biotech, Beijing, China). All the qPCR primer pairs (for primer sequences, see Table S1, see online supplementary material) displayed similar amplification efficiency (90–110%). Relative quantities were normalized against the geometric mean of the reference genes *VvACTIN* and *VvGAPDH* using the 2^-δδCt^ method [66].

### Phylogenetic analysis, VabHLH137 subcellular localization in tobacco, and transactivation assay

The conserved domain of VAG0123150 (VabHLH137) was analysed using the NCBI Conserved Domain Database (CDD, https://www.ncbi.nlm.nih.gov/cdd). Multiple sequence alignments were performed using the Clustal W program. The protein sequences of the bHLH TFs for *Arabidopsis thaliana* were downloaded from the *EnsemblPlants* database (http://plants.ensembl.org/index.html). The reported grapevine bHLH TFs were obtained from the NCBI database (https://www.ncbi.nlm.nih.gov). The phylogenetic analysis was conducted using the neighbor-joining method in the MEGA 11 software with bootstrap values of 1000 replicates.

The full-length coding sequence (CDS) of *VabHLH137* was amplified using PrimeSTAR® Max DNA polymerase (Takara, Osaka, Japan) and then ligated into pEAQ-GFP vectors to produce the fusion construct VabHLH137-GFP using a ClonExpress II One Step Cloning Kit (Vazyme, Nanjing, China). The sequenced plasmid was introduced into the tobacco leaves via *Agrobacterium tumefaciens* GV3101 strain infiltration, while an empty vector was used as a control. The GFP fluorescence in the tobacco (*N. benthamiana*) leaves was observed after incubation for 2–3 days using a confocal laser scanning microscope (Leica TCS SP5, Wetzlar, Germany).

To investigate the transcriptional activity of VabHLH137, pGBKT7-VabHLH137 and pGBKT7 (negative control) were transformed into yeast Y2H Gold strain. Transformants were cultivated on the corresponding medium at 30°C for 3–4 d.

### Yeast one-hybrid assays on the grape calli

The CDS of *VabHLH137* was ligated into the pB42AD vector to generate the AD-VabHLH137 construct. The *VaLAR2* promoter was cloned into the pLacZi2μ vector to yield the *VaLAR2p: LacZ* reporter constructs driving *LacZ* gene expression. The subsequent plasmids were co-transformed into the yeast EGY48 strain. The interactions were examined on a medium without Trp and Ura (SD/−Trp/-Ura), containing 5-bromo-4-chloro-3-indolyl-*β*-D-galactopyranoside (X-Gal) for blue color development [67].

### Electrophoretic mobility shift assay

The full-length CDS of *VabHLH137* without the stop codon was inserted into the pGEX4T-1 vector to construct a recombinant plasmid for VabHLH137-GST protein expression in Rosetta (DE3) *Escherichia coli*. The soluble recombinant VabHLH137-GST protein was purified with Glutathione Sepharose 4B (GE Healthcare, Chicago, IL, USA) [68]. The probe containing the E-box (CACATG) was labeled with biotin. EMSA was conducted according to the instructions of the manufacturer (Thermo Scientific, Waltham, MA, USA).

### Grapevine transformation

The CDS of *VabHLH137* was recombined into the pBI221 plasmid harboring the CaMV 35S promoter to generate VabHLH137-OE. Furthermore, the 300 bp sense and antisense sequences of *VabHLH137* were amplified using the gene-specific primers to generate RNA interference constructs. Both fragments were cloned into the pRNAi vector to obtain VabHLH137-RNAi.

The recombined plasmids were individually transformed into *A. tumefaciens* strains GV3101 and then transferred into the Cabernet Sauvignon grape skin calli. The putative transgenic calli were selected on B5 solid medium containing 4 mg/L hygromycin B. The primers used are listed in Table S1, see online supplementary material.

### Determination of the PAs in the grapevine calli

The PAs in the calli were detected and stained using DMACA reagent [6 M HCl: 0.2% DMACA (w/v) in methanol, 1:1 (v/v)] [37, 69]. Briefly, 0.5 g of the calli were ground in liquid nitrogen, and the frozen powder was dissolved in 1 mL of a 70% (v/v) aqueous acetone solution with 0.1% (w/v) ascorbic acid. The mixture was briefly vortexed and extracted at 4°C in the dark for 1 h. The procedure was repeated three times in the same conditions, while the supernatant was collected via centrifugation. Then, 200 μL of the supernatant was incubated with 0.2% DMACA at room temperature for 20 min. Finally, the PAs were quantified by measuring the absorbance of the mixture at 643 nm. The results were calculated via a standard curve prepared using (+)-catechin (Sigma, St. Louis, MO, USA).

### Pathogen infection assays of the grape calli

Briefly, WT and transgenic grape calli of the same weight were transferred to a B5 solid medium (without any antibiotic). After culturing in the dark at 25°C for 3 d, the calli were inoculated with 0.5-cm-diameter agar discs containing *C. gloeosporioides* mycelia and incubated for another 5 d at 25°C in the dark. Each experimental treatment was performed in triplicate, and the experiments were repeated at least three times. The diameters of the diseased locations of each sample were measured to obtain the average value of the three measurements.

### Yeast two-hybrid assays

The Y2H experiments were performed according to the instructions of the manufacturer (Clontech, Palo Alto, CA, USA). The C-terminal deletion versions of VabHLH137 (VabHLH137^ΔC^, amino acids 1–266) and the full-length cDNA of *VaMYBPAR* were inserted into pGBKT7 and pGADT7 to construct BD-VabHLH137^ΔC^ and AD-VaMYBPAR. The subsequent plasmids were co-transformed into Y2H Gold yeast strains using the PEG/LiAC method. The transformants were then screened on the selection medium supplemented with SD base/−Trp/−Leu/-His/−Ade in the presence of 5-bromo-4-chloro-3-indolyl-α-D-galactopyranoside (X-α-Gal) to test for interaction.

### Bimolecular fluorescence complementation assay

A BiFC assay was used to detect the interaction *in vivo*. The CDS of *VabHLH137* without a termination codon was inserted into the pSPYCE-35S plasmid to generate the VabHLH137-YFP^C^ construct, while VaMYBPAR without the stop codons was cloned into the pSPYNE-35S plasmid to generate the VaMYBPAR-YFP^N^ construct. The recombinant plasmids were transformed into *A. tumefaciens* GV3101 strains and then infiltrated into 5- to 6-week-old tobacco leaves. After infiltration 48 h, the YFP fluorescence in the tobacco cells was imaged using a confocal microscope (Leica TCS SP5, Wetzlar, Germany).

### Coimmunoprecipitation analysis

The CDS of *VabHLH37* and *VaMYBPAR* without the stop codon were cloned into the pEAQ-HT and pEAQ-GFP vectors to generate the VabHLH137-His and VaMYBPAR-GFP constructs, respectively. *A. tumefaciens* cells containing VabHLH137-His were co-transformed with VaMYBPAR-GFP into the tobacco leaves. The CoIP was performed as described previously [70]. Briefly, the total protein was extracted from the tobacco leaves after incubation for 72-h, after which it was incubated overnight with an anti-GFP antibody (Abcam, Cambridge, UK) at 4°C. The input protein and immunoprecipitates were detected via immunoblotting with either anti-GFP or anti-His antibodies (Abcam, Cambridge, UK).

### Dual-luciferase reporter assays

A transient expression assay was performed was performed in the tobacco (*N. benthamiana*) leaves using *Agrobacterium*-infiltration based on a previous description [70]. A 2 kb sequence of the *VaLAR2* promoter was cloned into the pGreenII 0800-LUC vector to produce the reporter, while the The CDS of *VabHLH137* was inserted into the pEAQ vector as an effector. The empty pEAQ vector was used as the negative control. The resulting vectors were transferred separately into *A. tumefaciens* GV3101 strains. The *A. tumefaciens* containing the reporter and effector constructs were co-transfected into the tobacco leaves. After 72 h of infiltration, the LUC signal of the infiltrated leaves was detected using the Tanon 5200 Multi Automatic chemiluminescence image analysis system (Tanon, Shanghai, China). The firefly luciferase (LUC) and *Renilla* luciferase (REN) activity were quantified using a Dual-Luciferase Reporter Assay Kit (Vazyme, Nanjing, China). The relative luciferase activity was calculated as the ratio of LUC/REN. Six biological repeats were measured for each sample.

### Accession numbers

Sequence data from this article can be found in the Genbank data library (https://www.ncbi.nlm.nih.gov/genbank/) using the following accession numbers: VabHLH137: UMB20802, VaMYBPAR: MW753047, VaMYBPA1: MW753049, VvMYC1: NP_001268182, VvMYCA1: ABM92332, VabHLH1: AFH68208.1, VvbHLH93: XP_002264407.

## Acknowledgements

We thank Prof. Daqi Fu, Prof. Benzhong Zhu, Prof. Junping Gao (China Agricultural University) and Prof. Jianye Chen (South China Agricultural University) for providing vectors; and Dr Yi Wang (IBCAS) for help with transcriptome data analysis. This project was supported by the National Natural Science Foundation of China (No. 31171942 and No. 31471835).

## Author Contributions

J.Z. conceived of the project, designed the experiments and supervised the research. D.Y. contributed to the experimental designs, performed experiments, analysed the results, and wrote the manuscript. W.W. and Z.F. performed EMSA and CoIP experiments. J.C. provided plasmids and experimental assistance. Y.Y. and W.H. discussed and reviewed the manuscript.

## Data availability

The authors confirm that all data that are needed to replicate this study and to draw conclusions are within the paper.

## Conflict of interests

The authors declare no conflict of interest.

## Supplementary data

Supplementary data is available at *Horticulture Research* online.

## Supplementary Material

Web_Material_uhac261
