# Supplementary figures and images for: VabHLH137 promotes proanthocyanidin and anthocyanin biosynthesis and enhances resistance to Colletotrichum gloeosporioides in grapevine
Source: Hortic Res. 2022 Dec 2;10(2):uhac261. doi: 10.1093/hr/uhac261 (PMC9907051; doi:10.1093/hr/uhac261)

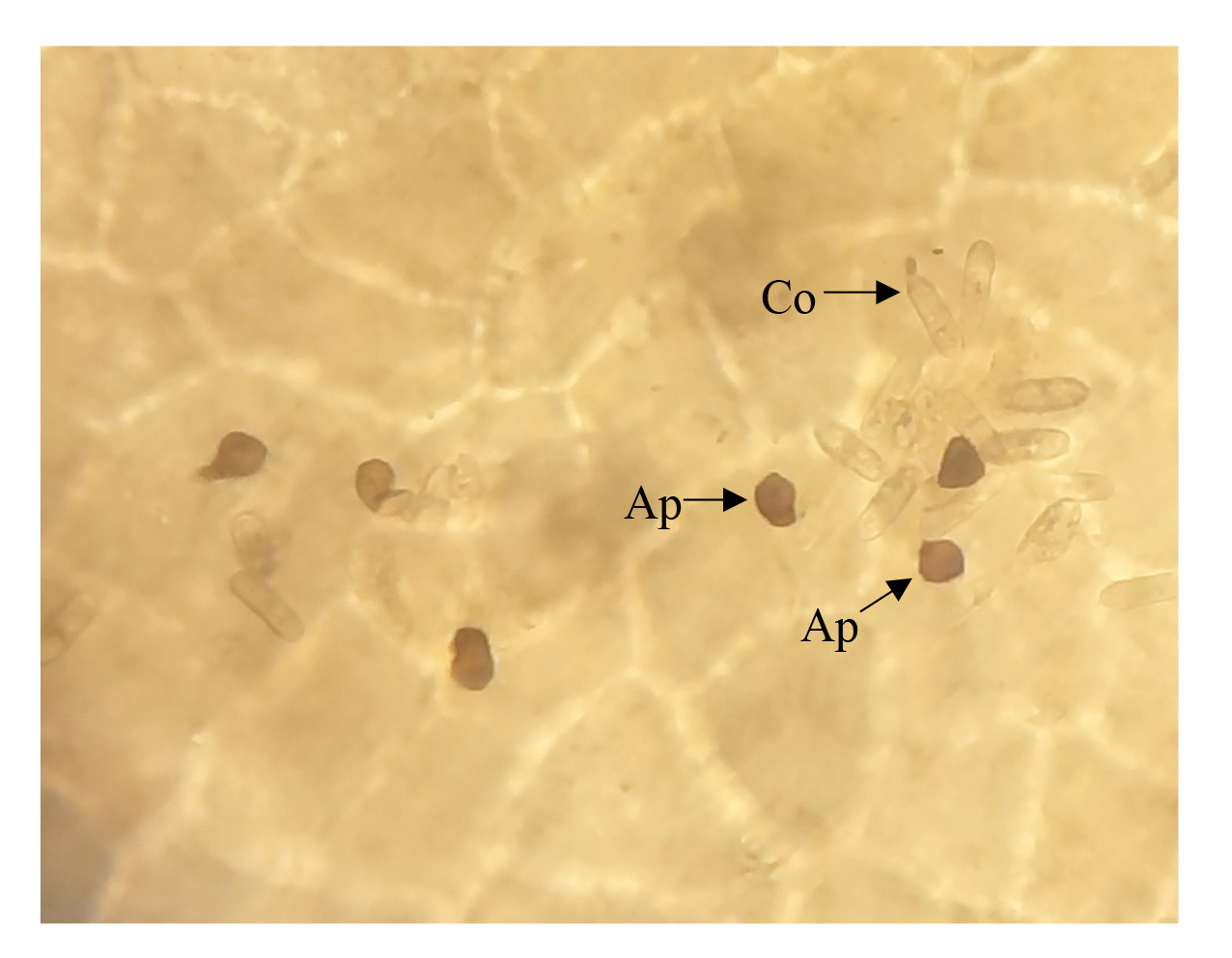

Supplement: Web_Material_uhac261 [file web_material_uhac261.zip › Figure S1.tif]

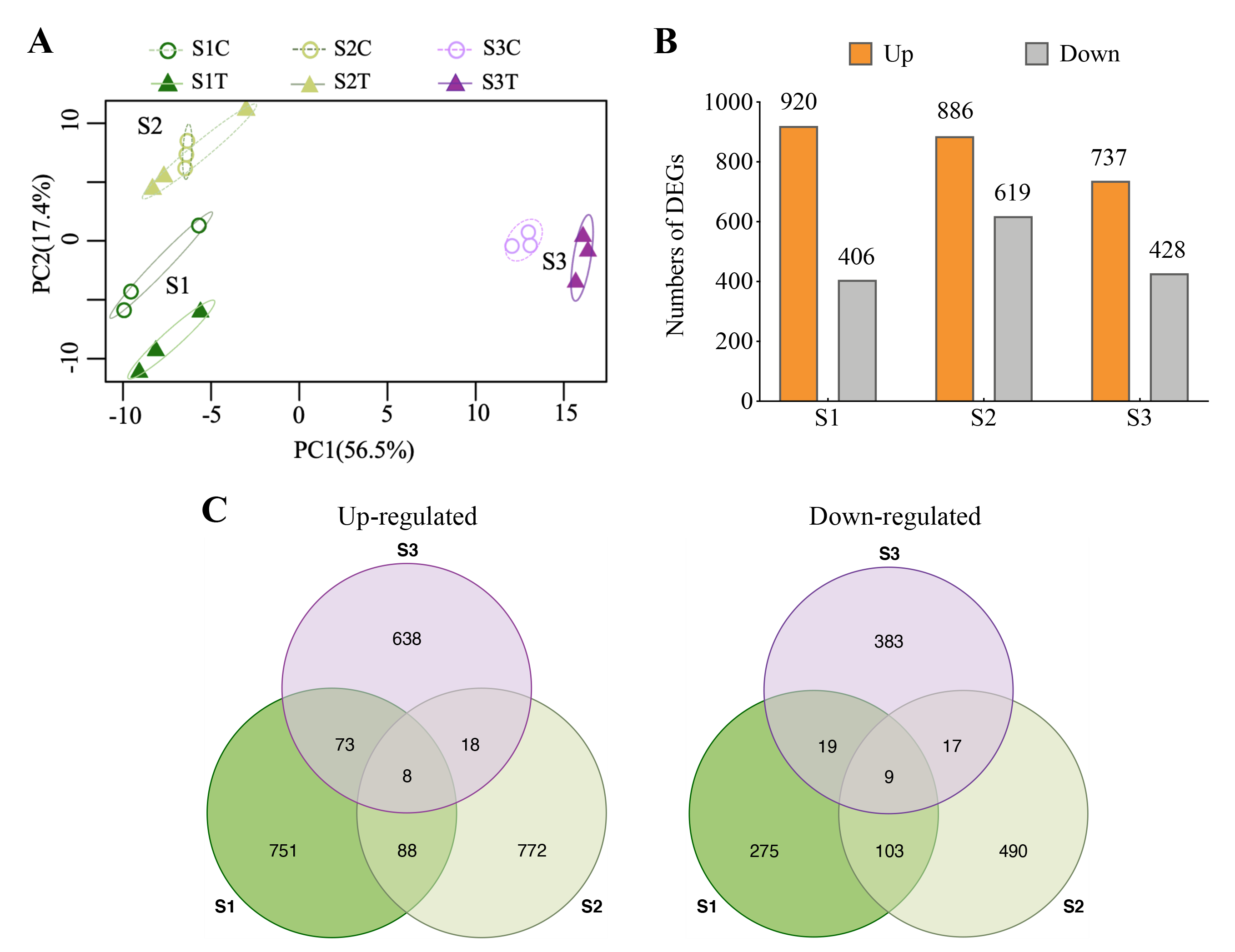

Supplement: Web_Material_uhac261 [file web_material_uhac261.zip › Figure S2.tif]

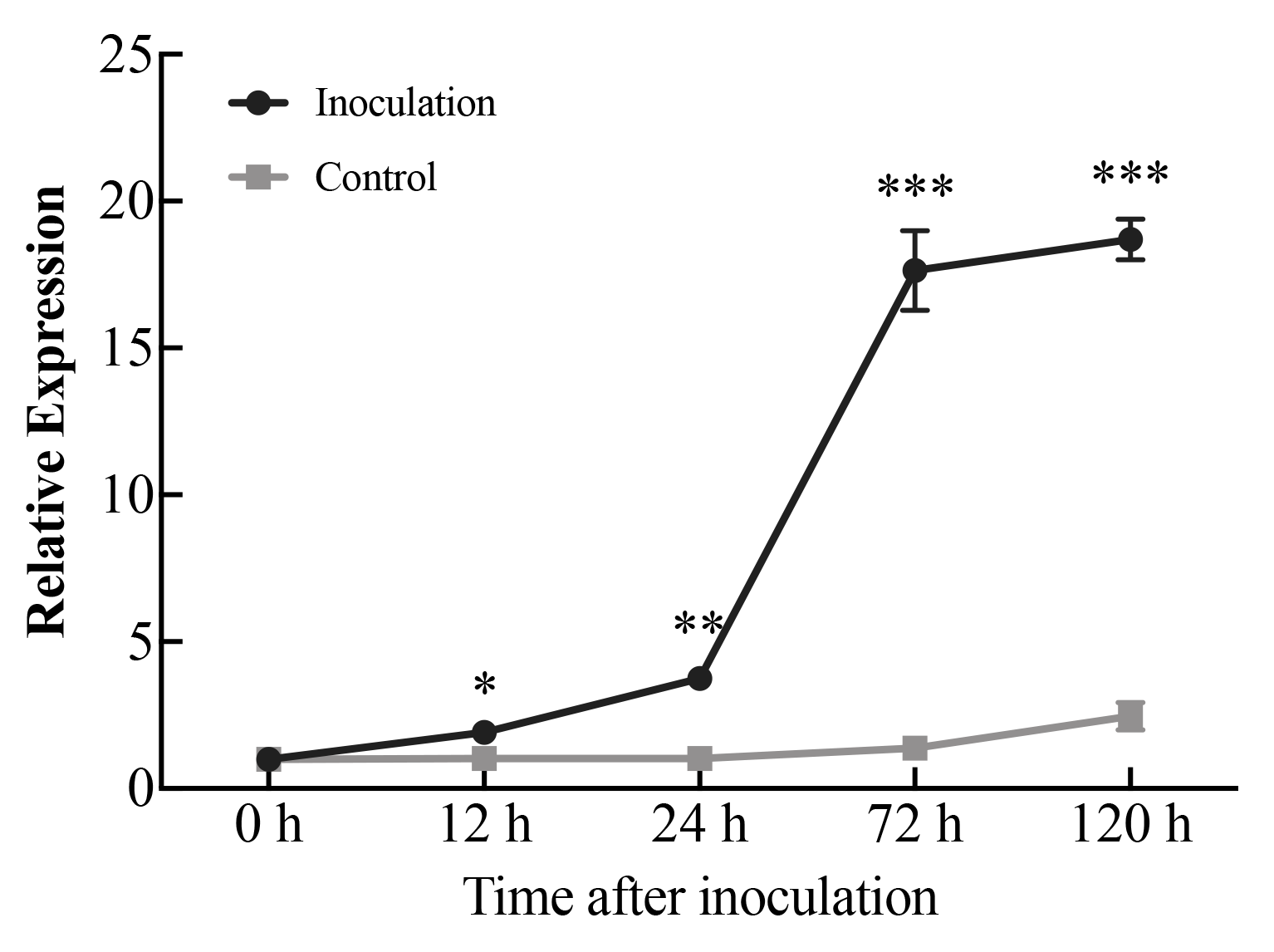

Supplement: Web_Material_uhac261 [file web_material_uhac261.zip › Figure S3.tif]

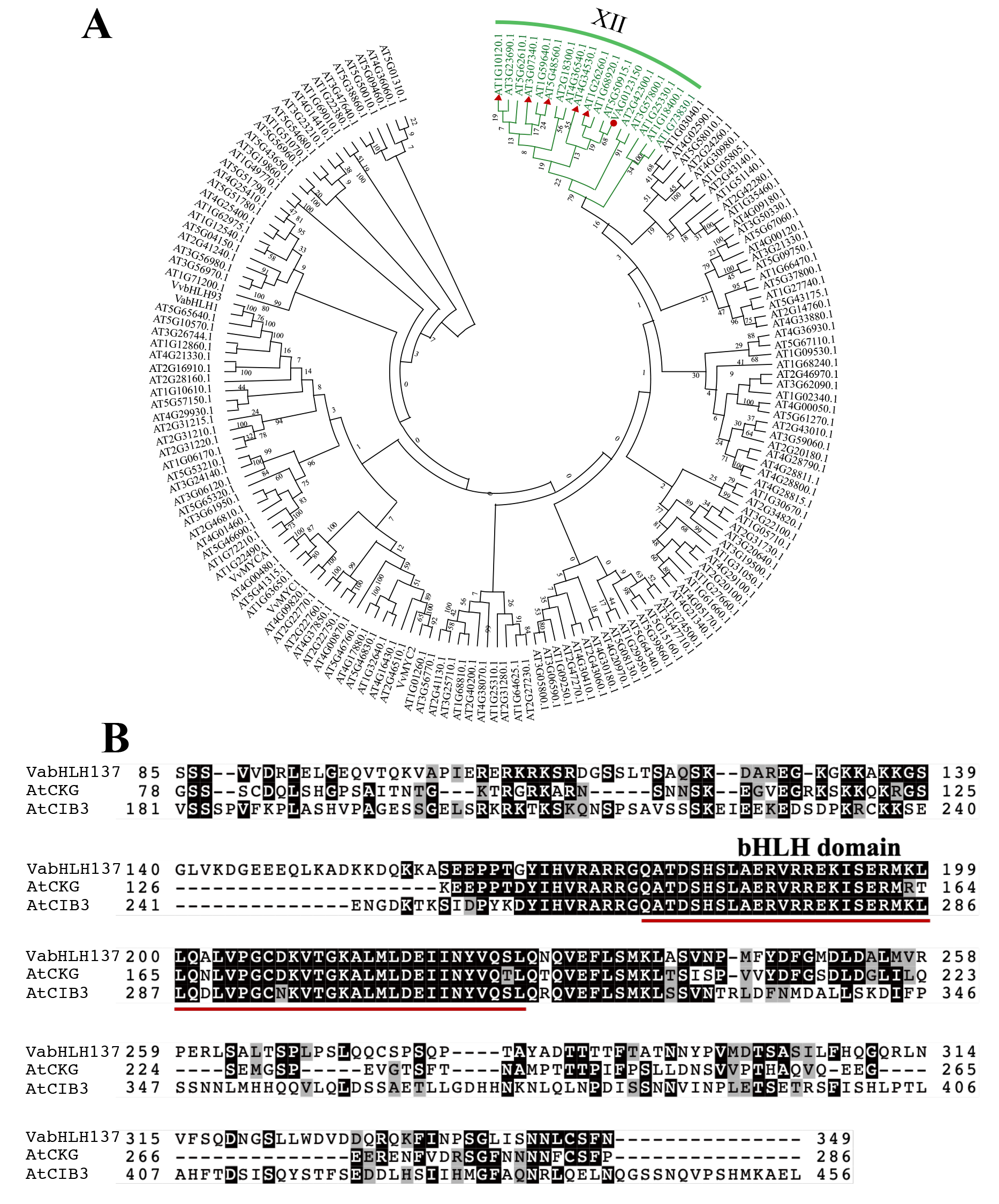

Supplement: Web_Material_uhac261 [file web_material_uhac261.zip › Figure S4.tif]

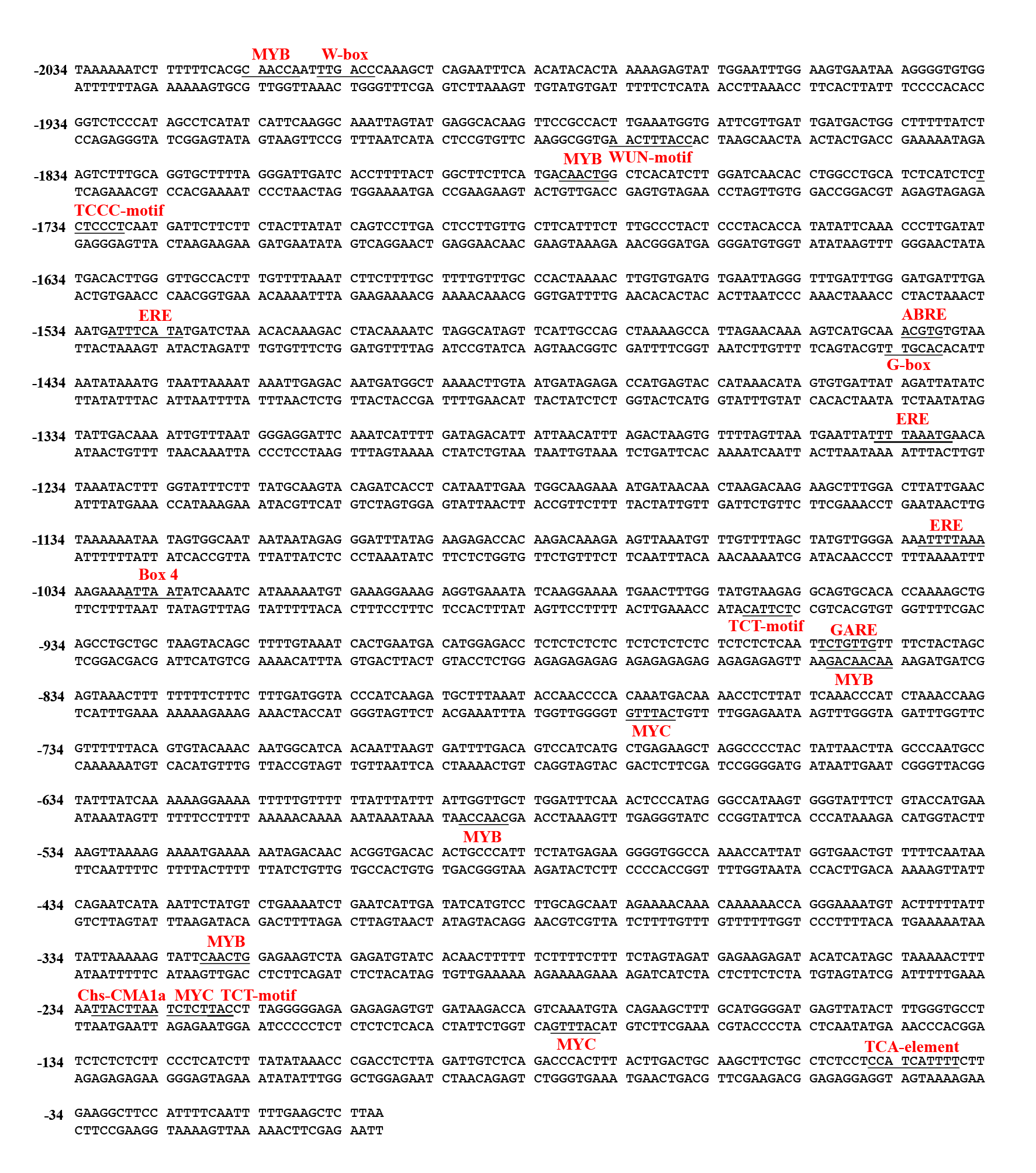

Supplement: Web_Material_uhac261 [file web_material_uhac261.zip › Figure S5.tif]
