# Supplementary material for: VabHLH137 promotes proanthocyanidin and anthocyanin biosynthesis and enhances resistance to Colletotrichum gloeosporioides in grapevine
Source: Hortic Res. 2022 Dec 2;10(2):uhac261. doi: 10.1093/hr/uhac261 (PMC9907051; doi:10.1093/hr/uhac261)
Supplement: Web_Material_uhac261 [file web_material_uhac261.zip › Additional document.doc]

**SUPPLEMENTARY FIGURES**

**Supplemental Figure S1. Optical microscopy images of the melanized appressoria on the green grape berry skin.** On the grape berry at EL33 stage, the *Colletotrichum gloeosporioides* becomes quiescent within 120 hpi as melanized appressoria. Co, conidia; Ap, appressoria.

**Supplemental Figure S2. Analysis of the RNA-seq data and DEGs**

(**A**) The PCA displaying the biological variations among the samples. S1 (infection initiation, 24 hpi of EL33), S2 (quiescent stage, 120 hpi of EL33), and S3 (necrotrophic stage, 96 hpi of EL35); C, sample control; T, *C. gloeosporioides* inoculated. (**B**) The number of DEGs (log_2_ fold change ratio > 1, *q*-values < 0.05) after *C. gloeosporioides* infection during the S1, S2, and S3 stages. (**C**) A Venn diagram showing the number of up- (left) or down-regulated (right) DEGs in response to *C. gloeosporioides* during the S1, S2, and S3 stages.

**Supplemental Figure S3. Expression of *VaMYBPA1* in *Vitis amurensis* in response to *C. gloeosporioides*.** *VvACTIN* and *VvGAPDH* genes were used for data normalization. Error bars represent the mean ± SD of three independent replicates. The statistical significance was determined using Student’s t-test (***P* < 0.05; ***P* < 0.01; ****P* < 0.001).

**Supplemental Figure S4. Sequence analysis of VabHLH37**

(**A**) Phylogenetic tree of VabHLH137, the reported flavonoid-related bHLHs in grapevine and all bHLH TFs from *Arabidopsis thaliana*. The bar indicates subgroup XII. The circle represents VabHLH137 and the triangles represent *Arabidopsis* CIBs. (**B**) Protein sequence alignment of VabHLH137, AtCKG (AtbHLH137) and AtCIB3. The bHLH domain is underlined in red.

**Supplemental Figure S5. Plant *cis*-acting regulatory elements in the *VabHLH137* promoter.** The motifs are labeled by lines in the sequence of *VabHLH13* promoter. The numbers on the left represent the numbers of the nucleotides upstream from the start point.

**SUPPLEMENTARY TABLES**

Supplemental Table S1. Primer sequences used in the present study.

Supplemental Table S2. Transcriptome data of DEGs.

Supplemental Table S3. Transcriptome data of KEGG classifications.

Supplemental Table S4. Transcriptome data of GO enrichment.

Supplemental Table S5. Selected transcription factors.
